# Flexible, transparent electrodes using carbon nanotubes

**DOI:** 10.1186/1556-276X-7-571

**Published:** 2012-10-17

**Authors:** Núria Ferrer-Anglada, Jordi Pérez-Puigdemont, Jordi Figueras, Muhammad Zahir Iqbal, Siegmar Roth

**Affiliations:** 1 Applied Physics Department, Universitat Politècnica de Catalunya, Campus Nord B4, J Girona 1-3, Barcelona, Catalonia, 08034, Spain; 2 Max Planck Institute for Solid State Research, Heisenbergtrasse 1, Stuttgart, 70569, Germany; 3 Department of Physics and Graphene Research Institute, Sejong University, Seoul, 143-747, South Korea; 4 School of Electrical Engineering, WCU Flexible Nanosystems, Korea University, Seoul, 136-713, South Korea

**Keywords:** Flexible, Transparent electrodes, Transparent conducting thin films, Flexible conducting films, Raman, Electrical impedance

## Abstract

We prepare thin single-walled carbon nanotube networks on a transparent and flexible substrate with different densities, using a very simple spray method. We measure the electric impedance at different frequencies *Z*(*f*) in the frequency range of 40 Hz to 20 GHz using two different methods: a two-probe method in the range up to 110 MHz and a coaxial (Corbino) method in the range of 10 MHz to 20 GHz. We measure the optical absorption and electrical conductivity in order to optimize the conditions for obtaining optimum performance films with both high electrical conductivity and transparency. We observe a square resistance of 1 to 8.5 kΩ for samples showing 65% to 85% optical transmittance, respectively. For some applications, we need flexibility and not transparency: for this purpose, we deposit a thick film of single-walled carbon nanotubes on a flexible silicone substrate by spray method from an aqueous suspension of carbon nanotubes in a surfactant (sodium dodecyl sulphate), thereby obtaining a flexible conducting electrode showing an electrical resistance as low as 200 Ω/sq. When stretching up to 10% and 20%, the electrical resistance increases slightly, recovering the initial value for small elongations up to 10%. We analyze the stretched and unstretched samples by Raman spectroscopy and observe that the breathing mode on the Raman spectra is highly sensitive to stretching. The high-energy Raman modes do not change, which indicates that no defects are introduced when stretching. Using this method, flexible conducting films that may be transparent are obtained just by employing a very simple spray method and can be deposited on any type or shape of surface.

## Background

Flexible conducting thin films are useful in flexible electronic devices for different applications such as sensors, transistors, or flexible electrodes. Transparent, flexible electrodes could be used in photovoltaic solar cells as an alternative material to conducting oxides as ITO.

Based on single-walled carbon nanotubes (SWCNTs), it is possible to make transparent, flexible networks with randomly distributed SWCNTs, either self-standing or on a transparent, flexible substrate [1-4] using low-cost methods, at low temperature and a non-vacuum process. Different research groups have obtained and studied the performances of flexible electrodes and transistors based on carbon nanotubes [5-8]. In order to obtain reproducible devices with an efficient performance, we need to characterize the fundamental properties of thin-film SWCNT networks.

With this objective, in this paper, we present our results in two different series of samples:

Part 1: We measure the frequency-dependent electrical impedance on transparent, flexible SWCNT networks by varying the SWCNT density and using different sample geometries up to 20 GHz. We analyze the cutoff frequency (*f*_0_) dependence on carbon nanotube density. After measuring the optical transmittance (%*T*), we can correlate the transmittance *T* with impedance.

Part 2: For some applications, we need flexibility rather than transparency: we obtain a dense network of SWCNTs on silicone and analyze the electrical conductivity behavior and Raman spectra when stretching the sample up to 20% elongations.

## Methods

The spray coating technique is the simplest and quickest method for depositing CNTs on a surface. Using a commercial airbrush (from Harder & Steenbeck, Norderstedt, Germany), one can easily tune the transparency of the samples from 0% to 100%. Thin films are prepared by spraying a suspension of carbon nanotubes (obtained by laser ablation at MPI f.k.f., Stuttgart, Germany) in an aqueous solution at 1% sodium dodecyl sulphate (SDS), after sonicating it for 1 h at 40 W, as described in [6], on a flexible substrate, polypropylene carbonate, for the transparent films. The density, film thickness, and transparency are controlled by the SWCNT concentration in SDS and the number of spray passes over the substrate, which is from three to ten in our samples: as the number of spray passes increases, the density also increases and the transparency evidently decreases. After the deposition of SWCNTs, the film is submerged in distilled water for 30 s and then dried in air. The flexible, nontransparent films were obtained in the same way: increasing the number of spray passes up to 20 to 40 and obtaining a dark, nontransparent film on a silicone substrate. A two-probe contact [2] and a coaxial (Corbino reflectometry) setup [3] are used for the electrical impedance measurements (Agilent E8362B and Agilent 4294A network analyzers, respectively, Agilent Technologies, Inc., Santa Clara, CA, USA). Raman spectra are recorded with a Jobin Yvon T64000 micro-Raman spectrometer (Horiba Ltd., Kyoto, Japan) coupled to an Olympus BH2 optical microscope (Olympus Corporation, Shinjuku-ku, Japan). The excitation line used is a 514.5-nm-wavelength argon laser under ambient conditions. The polarized Raman measurements are also obtained with the microscope in the backscattering configuration. Optical absorption spectroscopy is performed for thin film networks with different SWCNT densities in order to use the % *T* as a quantitative parameter proportional to the CNT density. The quantitative SWCNT amount can be deduced from the optical absorption spectra in the NIR range, in particular from the S_22_ peak, assigned to the second Van Hove transition [9]. In a previous paper, we show that this method is in good agreement with a TGA determination of the SWCNT amount, which is proportional to the % *T*[2].

## Results and discussion

### Part 1: Transparent, flexible SWCNT networks

**Figure 1:**
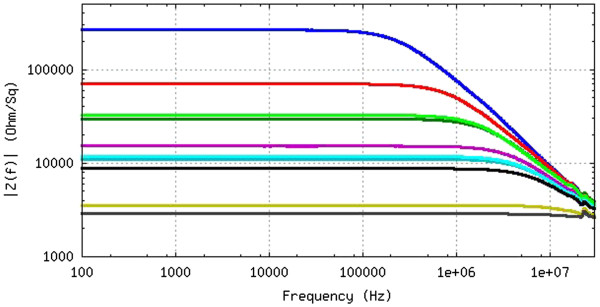
**Impedance dependence on frequency *Z*(*f*) for films of different SWCNT densities corresponding to different colors.** The highest impedance corresponds to the lowest density. We can define the cutoff frequency (*f*_0_), at which *Z* decreases abruptly: *f*_0_ increases when increasing the SWCNT density on the substrate, while resistance decreases, as does the low-frequency impedance.

**Figure 2:**
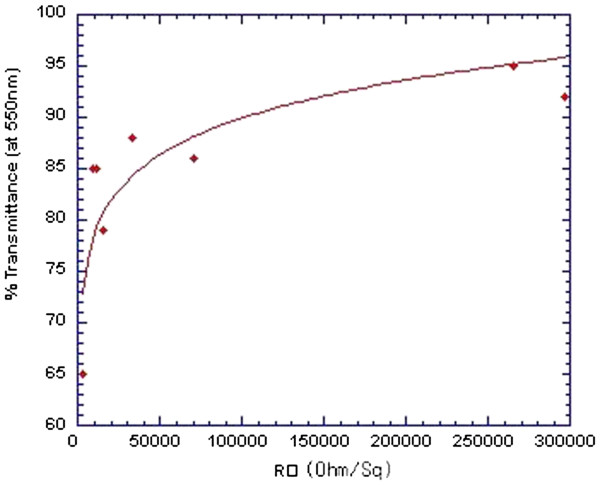
**Transparency (%*T *at 550 nm) versus resistance/square (*R*□) for networks of different carbon nanotube densities.** Red diamonds are the experimental points.

With this objective, we prepare thin SWCNT networks on a transparent and flexible substrate with different carbon nanotube densities, as described above. We measure the electrical impedance at different frequencies *Z*(*f*) in the frequency range of 40 Hz to 20 GHz using two different methods: a two-probe method in the range up to 110 MHz and a coaxial (Corbino) method in the range of 10 MHz to 20 GHz. Figure 1 shows how the impedance decreases with the increase in nanotube density while the cutoff frequency, defined as the frequency at which the resistance decreases abruptly, increases. This fact is demonstrated and commented on by other researchers [3]. We measure the optical absorption and the % *T* values, assuming that they are strictly dependent on the SWCNT density on the network. Figure 2 shows the correlation between transparency (% *T*) and electrical resistance. In order to optimize the conditions for optimum performance, films with both high electrical conductivity and transparency are desired. We observe a square resistance from 1.0 to 8.5 kΩ for samples showing 65% to 85% optical transmittance, respectively.

### Part 2: Flexible, dense SWCNT networks

The films are chemically and morphologically stable (either the conductivity or the Raman were reproducible for more than 1 year after multiple elongations) and quite uniform. The electrical resistance is as low as 200 Ω/sq and does not depend on frequency. When stretching the sample up to 10% elongation, the resistance increases by 11%, recovering its initial value after the elongation. When stretching is 20%, the resistance increases by 16%, recovering only partially its initial value.

**Figure 3:**
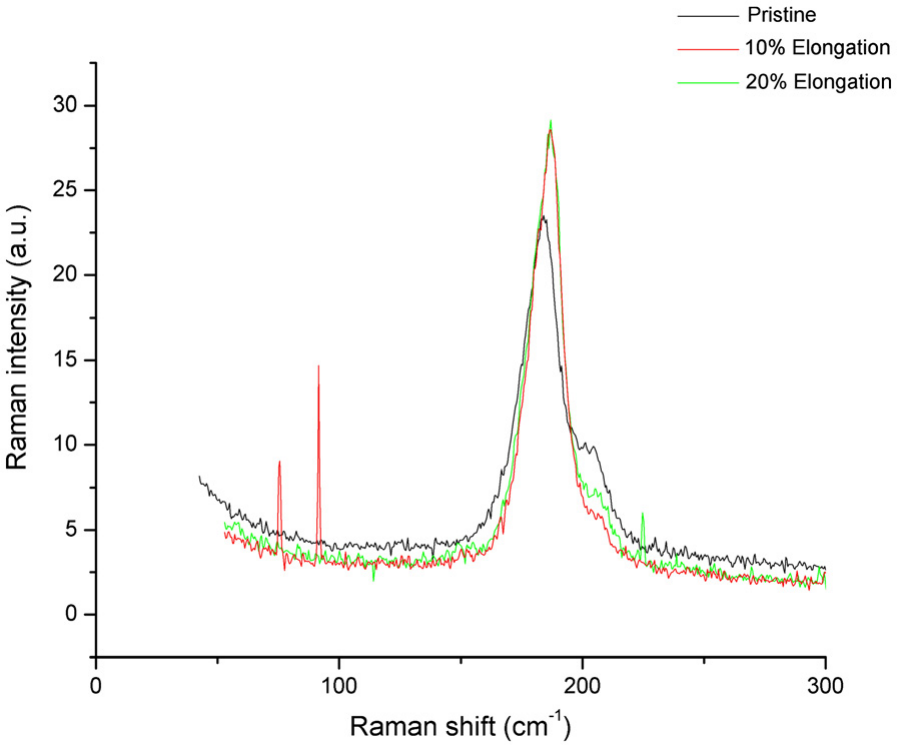
**Raman spectroscopy on the unstretched (pristine) and stretched samples.** It shows a clear shift on the breathing mode.

**Figure 4:**
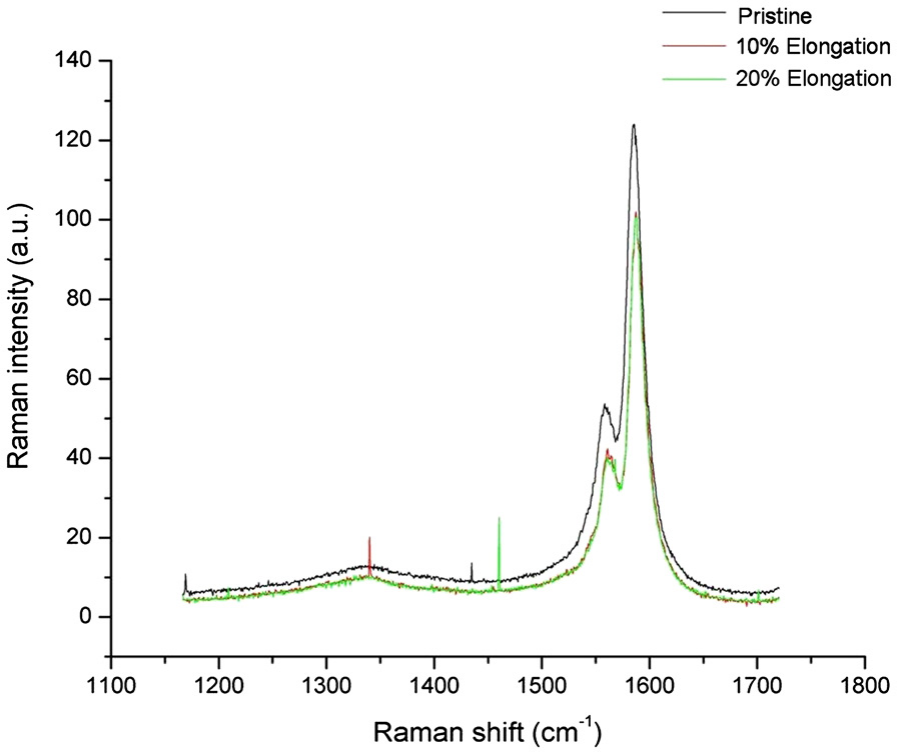
**Raman G (≈1,600 cm**^**−1**^**) and D (≈1,300 cm**^**−1**^**) lines remain virtually unchanged with/without stretching.**

**Figure 5:**
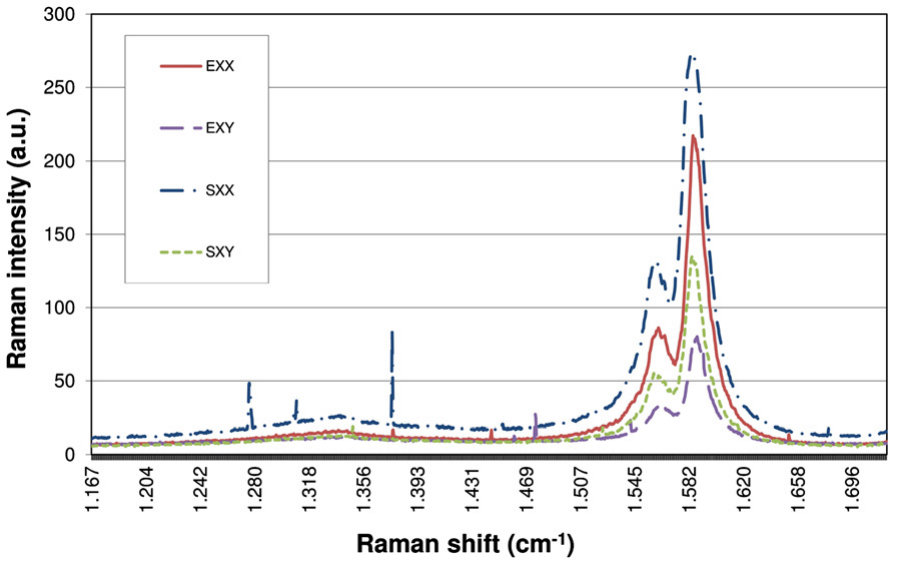
**Polarized-light Raman spectra recorded at two different perpendicular polarizations, XX and XY.** The Raman G line spectra SXX and SXY correspond to the unstretched samples; EXX and EXY, to stretched samples with 10% elongation. One may observe how the intensity of the relationship after elongation, EXX/EXY ≈ 2.76, is increased respectively similar to that before elongation, SXX/SXY ≈ 2.03, which indicates a preferred orientation by stretching. Furthermore, the relation D/G remains unchanged, indicating that stretching does not appreciably introduce defects.

Raman spectroscopy is performed using low power (0.3 mW) in order to obtain reproducible spectra, focusing on different points on two different samples: the unstretched and stretched samples; the comparison of spectra shows interesting general features: for example, in Figure 3, one may observe how the radial breathing mode appears at higher energies for the stretched samples. Figures 4 and 5 show that the G (around 1,600 cm^−1^) and D (1,300 cm^−1^) lines remain practically unchanged by elongation. It is well known that the intensity ratio of lines D/G is indicative of the CNT defects [10]. From these figures, we can see that the intensity ratios of D/G are not increased by stretching, indicating that stretching does not introduce appreciable defects. Figure 5 shows the Raman shift using polarized laser light, recorded at two different perpendicular polarizations, named XX and XY. Spectra SXX and SXY correspond to the unstretched samples, while EXX and EXY correspond to the stretched samples with 10% elongation. This figure (using the same arbitrary units of intensity) shows that the intensity of the relationship after elongation, EXX/EXY ≈ 2.76, is increased respectively similar to that before elongation, SXX/SXY ≈ 2.03, indicating a preferred orientation by stretching. Note that we did not record multipoint measurements in obtaining Raman mapping [10], but we recorded Raman spectra on different points and samples. Probably by 20% stretching, some SWCNTs are damaged or broken, producing a reduction of the electrical conductivity, but this is not visible at the observed points.

## Conclusions

We study the frequency-dependent impedance measurements on transparent, flexible single-walled carbon nanotube networks over a large range of frequencies, from 40 Hz to 20 GHz. Optical absorption spectroscopy confirms that high transparency, from 65% to 95%, can be obtained by controlling the carbon nanotube density, the most transparent samples corresponding to low-density and less conducting films.

We measure the electrical resistance, stability, and reversibility of flexible, dense single-walled carbon nanotube networks by stretching and Raman spectroscopy. The electrical square resistance is as low as 200 Ω/sq. Stretching is reversible for elongations up to 10%. Electrical conductivity is slightly lower for the elongated samples, recovering the initial value after elongation.

On the Raman spectra, breathing modes are very sensitive to stretching. The high-energy Raman modes remain unchanged, indicating that no defects are introduced when stretching. Polarized Raman show a partial orientation of CNTs on the silicone substrate when stretching.

In both cases, the use of selected metallic carbon nanotubes could enhance the electrical conductivity by a factor of 5 to 10 [11]. Furthermore, prior carbon nanotube purification will enhance transparency, thereby increasing film performance. Recent results using graphene to obtain flexible, transparent electrodes are highly successful [12,13], the samples obtained showing sheet resistances as low as 25 to 125 Ω/sq for 90% to 97.4% transparency, respectively. However, in our case, flexible conducting films, which could be transparent, are obtained just by using a very simple spray method and can be deposited on any type and shape of surface.

## Competing interests

The authors declare that they have no competing interests.

## Authors’ contributions

NFA planned the second part of the work, planned and interpreted the Raman spectroscopy, and wrote the paper. JPP provided some of the samples and conducted the two-probe impedance measurements. JF made the dense film samples and the Raman spectroscopy. MZI measured and analyzed the impedances with the Corbino method and conducted the optical absorption spectroscopy. SR provided the carbon nanotubes, planned the first part of the work, and conducted discussions on different aspects and applications. All authors read and approved the final manuscript.
